# DNA polymerase hybrids derived from the family-B enzymes of *Pyrococcus furiosus* and *Thermococcus kodakarensis*: improving performance in the polymerase chain reaction

**DOI:** 10.3389/fmicb.2014.00224

**Published:** 2014-05-27

**Authors:** Ashraf M. Elshawadfy, Brian J. Keith, H'Ng Ee Ooi, Thomas Kinsman, Pauline Heslop, Bernard A. Connolly

**Affiliations:** Institute of Cell and Molecular Biosciences, University of NewcastleNewcastle upon Tyne, UK

**Keywords:** PCR, *Pyrococcus furiosus*, *Thermococcus kodakarensis*, archaeal DNA polymerase, domain swapping, thermostability, fidelity

## Abstract

The polymerase chain reaction (PCR) is widely applied across the biosciences, with archaeal Family-B DNA polymerases being preferred, due to their high thermostability and fidelity. The enzyme from *Pyrococcus furiosus* (Pfu-Pol) is more frequently used than the similar protein from *Thermococcus kodakarensis* (Tkod-Pol), despite the latter having better PCR performance. Here the two polymerases have been comprehensively compared, confirming that Tkod-Pol: (1) extends primer-templates more rapidly; (2) has higher processivity; (3) demonstrates superior performance in normal and real time PCR. However, Tkod-Pol is less thermostable than Pfu-Pol and both enzymes have equal fidelities. To understand the favorable properties of Tkod-Pol, hybrid proteins have been prepared. Single, double and triple mutations were used to site arginines, present at the “forked-point” (the junction of the exonuclease and polymerase channels) of Tkod-Pol, at the corresponding locations in Pfu-Pol, slightly improving PCR performance. The Pfu-Pol thumb domain, responsible for double-stranded DNA binding, has been entirely replaced with that from Tkod-Pol, again giving better PCR properties. Combining the “forked-point” and thumb swap mutations resulted in a marked increase in PCR capability, maintenance of high fidelity and retention of the superior thermostability associated with Pfu-Pol. However, even the arginine/thumb swap mutant falls short of Tkod-Pol in PCR, suggesting further improvement within the Pfu-Pol framework is attainable. The significance of this work is the observation that improvements in PCR performance are easily attainable by blending elements from closely related archaeal polymerases, an approach that may, in future, be extended by using more polymerases from these organisms.

## Introduction

**Figure 1:**
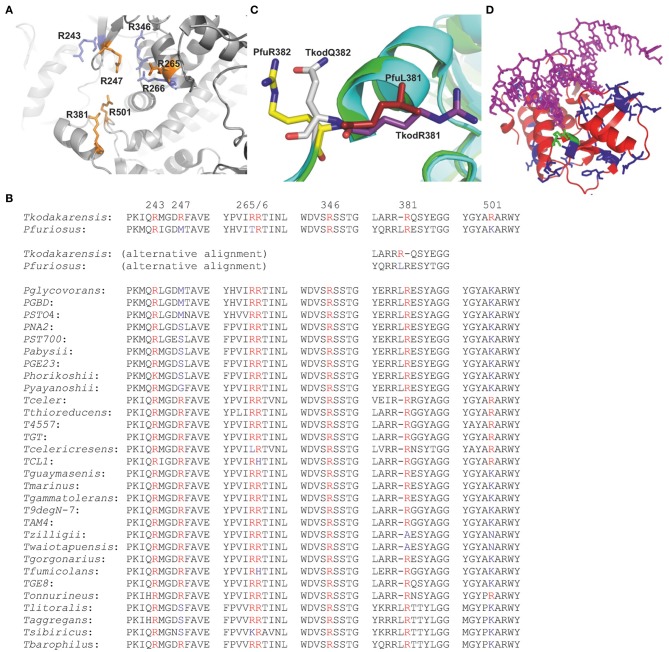
**“Forked-point” arginines in family-B DNA polymerases from the *Thermococcales* order of the archaea. (A)** Close spatial proximity of the forked-point arginines in Tkod-Pol (pdb; 1GCX). The arginines shown in orange are not conserved in Pfu-Pol and are the subject of this study. The arginines shown in blue are present in both Tkod-Pol and Pfu-Pol. **(B)** Amino acid sequence line up of the “forked-point” arginines and their immediate neighbors. The seven arginines present at the forked-point of *Thermococcus kodakarensis* DNA polymerase are shown in red and their position in the polypeptide chain indicated by the numbers above the sequence. Retention of arginine in other polymerases is indicated in red, change to an alternative amino acid is shown in blue. As discussed in the text, the insertion/deletion at amino acid 381 allows for two alternative alignments. For the species list, T = *Thermococcus*, P = *Pyrococcus*. **(C)** Superimposition of the Tkod-Pol (main chain in blue) (pdb; 1GCX) and Pfu-Pol (main chain in green) (pdb; 2JGU) near arginine 381. The overlay clearly shows that the spatial equivalent of Tkod-Pol R381 (purple) is Pfu-Pol L381 (red), rather than Pfu-Pol R382 (yellow) which is near Tkod-Pol Q382 (gray). **(D)** Thumb domain of Tkod-Pol bound to DNA in a polymerization mode (pdb; 4K8Z). The amino acids that are different in Pfu-Pol are shown in blue (no direct contact to DNA) or green (direct contact to DNA).

Thermostable DNA polymerases are essential components of the polymerase chain reaction (PCR), a technique with myriad uses across the life sciences (Mullis et al., 1994; Weissensteiner et al., 2004; McPherson and Möller, 2006; Saunders and Lee, 2013). The family-B DNA polymerases from the *Thermococcales* order of the archaea are especially favored for PCR, due to their extreme stability at elevated temperatures and the presence of fidelity-conferring 3′-5′ exonuclease activity (Lundberg et al., 1991; Cline et al., 1996; Takagi et al., 1997; Nishioka et al., 2001). Both the amino acid sequences and X-ray structures of these polymerases demonstrate a high degree of similarity, with structures available for the enzymes isolated from *Thermococcus gorgonarius* (Tgo-Pol) (Hopfner et al., 1999; Firbank et al., 2008; Killelea et al., 2010), *Thermococcus kodakarensis* (Tkod-Pol) (Hashimoto et al., 2001; Kuroita et al., 2005; Bergen et al., 2013), *Thermococcus* species 9°N-7 (9°N-Pol) (Chapin-Rodriguez et al., 2000), *Pyrococcus furiosus* (Pfu-Pol) (Kim et al., 2008) and *Pyrococcus abysii* (Pab-Pol) (Gougel et al., 2012). Despite similarities of amino acid sequence and structure, these polymerases have diverse kinetic properties; a strong influence on PCR performance. Most notably Tkod-Pol possesses higher processivity (the number of dNTPs incorporated per binding event) than other enzymes (Takagi et al., 1997; Nishioka et al., 2001). Enhanced processivity may arise from the presence of seven arginines, suggested to play a role in stabilizing primer-template binding and influencing the movement of DNA between the polymerization and proof reading active sites (Hashimoto et al., 2001; Kim et al., 2008). These arginines cluster near the “forked-point” (the junction between the template-binding and editing clefts) of Tkod-Pol (Figure 1A). The seven amino acids are well conserved in *Thermococcales* DNA polymerases, with two (R243 and R264) being present in all species (Figure 1B). The other five locations show more variation, although the amino acid at 266 is an arginine in both Tkod-Pol and Pfu-Pol. At the remaining four positions (247, 365, 381, 501), the arginines present in Tkod-Pol are replaced by an alternative amino acid. The situation at Tkod-Pol position 381 is slightly obscured by insertion of an additional leucine at position 381 in Pfu-Pol. This arrangement, seen with many of the *Thermococcales* DNA polymerases, leads to two possible sequence alignments (Figure 1B). The first, generated by the alignment algorithm Clustal (Sievers et al., 2011), lines up Tkod-Pol R381 with Pfu-Pol R382. However, superimposition of the structures of the two polymerases (Figure 1C) shows that R381 in Tkod-Pol and L381 in Pfu-Pol are spatially equivalent, suggesting the alternative alignment shown in Figure 1B (Hashimoto et al., 2001). Another region that may contribute to processivity is the thumb domain, responsible for binding double-stranded DNA (Firbank et al., 2008; Killelea et al., 2010; Gougel et al., 2012; Bergen et al., 2013). This domain grips DNA tightly and interacts with newly synthesized double-strands, implying an important role in DNA translocation. In the absence of DNA, the thumb shows high flexibility and stretches of amino acids are often invisible in apo-enzyme crystal structures. The overall fold of the thumb domain does not differ significantly between archaeal DNA polymerases; however, its location relative to other domains is quite variable and entire domain motion is observed on DNA binding. Although the amino acid sequences in this domain are similar overall, many changes to individual amino acids are seen when Pfu-Pol and Tkod-Pol are compared (Figure 1D; supplementary data, Figure S1). However, in the current absence of polymerase-DNA-dNTP ternary structures, it is not easy to correlate any differences in processivity between Pfu-Pol and Tkod-Pol with individual amino acids in the thumb region.

During archaeal replication processivity is facilitated by PCNA, a sliding clamp which completely encircles the DNA, while simultaneously binding to the polymerase (Ishino and Ishino, 2011). Polymerase dissociation from DNA is hindered and overall processivity is enhanced, leading to rapid copying of the genome. In the *thermococcales* PCNA is a homo-trimeric ring-shaped molecule and the ring must be opened to load the protein onto DNA. *In vivo*, loading of PCNA is carried out by RFC, an ATP-driven clamp loader (Ishino and Ishino, 2011). However, *in vitro*, e.g., during the PCR, PCNA is not usually present and copying of DNA relies on the intrinsic processivity of the polymerase. Occasionally PCNA has been added and has been reported to enhance the PCR capability of Tkod-Pol (Kitabayashi et al., 2002). A recent comprehensive study showed that native Pfu-PCNA only improved PCR if RFC was also present, presumably to facilitate loading of the clamp onto DNA (Ishino et al., 2012). However, two PCNA mutants with reduced ring stability where able to self-load and considerably improved the PCR performance of Pfu-Pol, in the absence of RFC. For biotechnology applications more processive polymerases have been generated by fusion with double-stranded DNA binding proteins such as helix-hairpin-helix motifs (Pavlov et al., 2002) and Sso7d (a thermostable protein from *Sulfolobus solfataricus*) (Wang et al., 2004); the latter giving significant improvement in many PCR applications. Elevated processivity leads to more rapid copying of DNA and a polymerase with this property should be better at the PCR i.e., generate product more rapidly or produce longer amplicons. As processivity, *in vivo*, is largely a function of the sliding clamp, there may be little evolutionary pressure on polymerases to maximize this function, allowing considerable scope for improvement. In this publication we have sought to rationalize the differences in processivity and PCR ability previously observed between Tkod-Pol and Pfu-Pol. Hybrid polymerases have been produced, mainly Pfu-Pol but containing amino acids/domains from Tkod-Pol suggested to contribute to processivity. Do these hybrids result in a transfer of processivity from Tkod-Pol to Pfu-Pol and improved PCR performance? A better comprehension of why two structurally similar DNA polymerases have different kinetic properties is intrinsically important and may also guide further rational mutagenesis.

## Materials and methods

### Protein purification and mutagenesis

Wild type Pfu-Pol B (gene inserted into pET17b) was purified as reported (Evans et al., 2000; Emptage et al., 2008). Wild type TKod-Pol B (present in pET21a, supplied by Dr. Zvi Kelman University of Maryland) was purified in an identical manner. The single, double and triple mutants of Pfu-Pol were produced using a QuickChange® site directed mutagenesis kit (Agilent-Stratagene, Stockport) with Velocity™ DNA polymerase (Bioline, London, UK). The genes encoding the Pfu-TKod thumb swap derivatives were prepared using overlap extension PCR (Warrens et al., 1997). All mutated genes were completely sequenced to ensure the presence of the desired mutation and an absence of changes elsewhere. All mutants were purified in the same manner as the wild type enzyme with SDS-PAGE and Coomassie Blue staining indicating a purity of >95%.

### Polymerase and exonuclease assays

**Figure 2:**
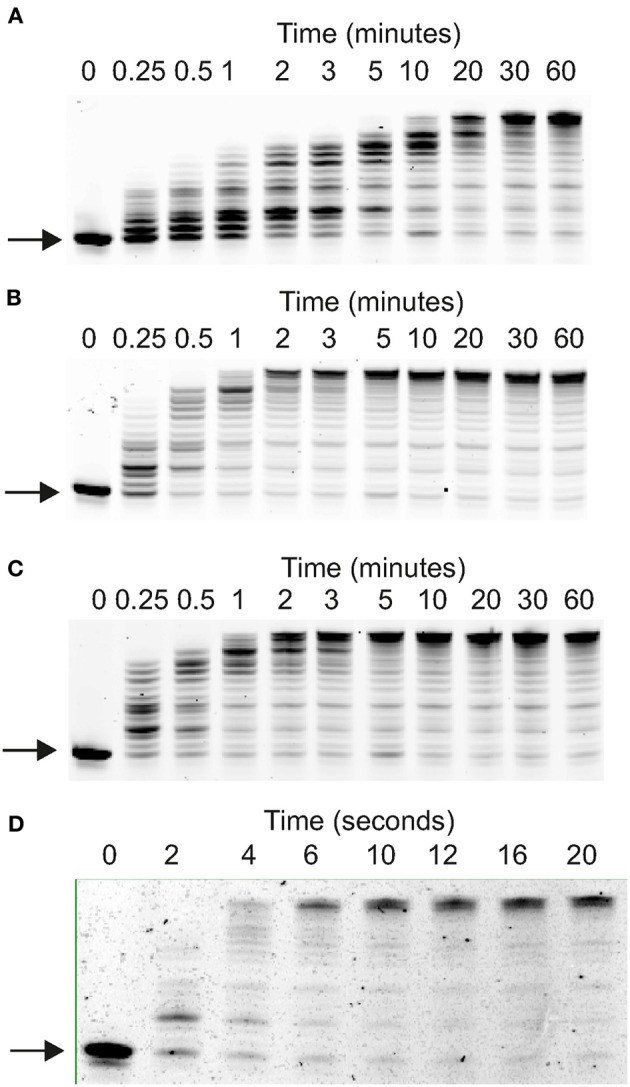
**Elongation of Primer-templates by archaeal family-B DNA polymerases**. Gel electrophoretic analysis of primer strand extension observed with: **(A)** Pfu-Pol wild type; **(B)** Pfu-Pol M247R/L381R; **(C)** Pfu-Pol L381R/K501R; **(D)** Tkod-Pol. The primer-template used was: 5′-GGGGATCCTCTAGAGTCGACCTGC 3′-CCCCTAGGAGATCTCAGCTGGACGACCGTTCGTTCGAACAGAGG. The primer was labeled at its 5′-terminus with either cyanine 5 (used with wild type Pfu-Pol and the double mutants) or fluorescein (used with Tkod-Pol).

**Figure 3:**
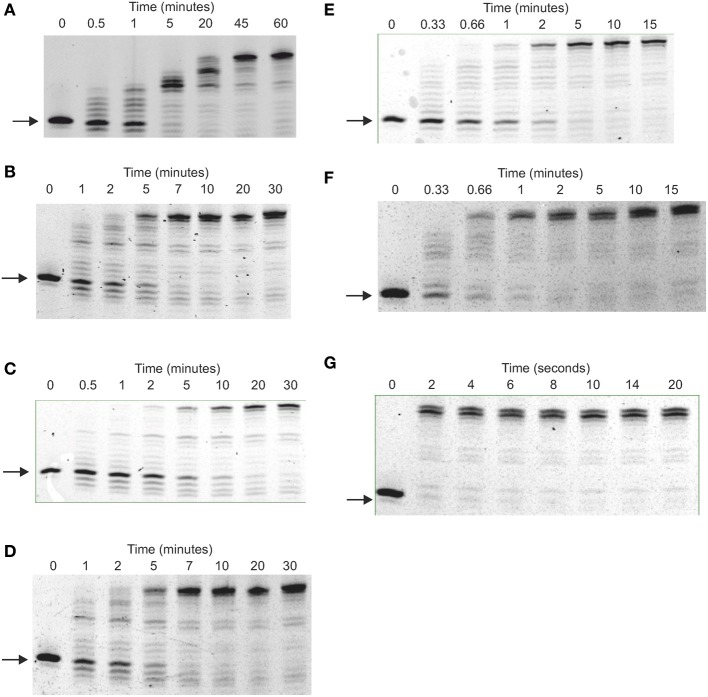
**Elongation of primer-templates by archaeal family-B DNA polymerases**. Gel electrophoretic analysis of primer strand extension observed with: **(A)** Pfu-Pol wild type; **(B)** Pfu-Pol M247/L381; **(C)** Pfu-Pol L381R/K501R; **(D)** Pfu-Pol M247R/L381R/K501R; **(E)** Pfu-TkodTS; **(F)** Pfu-TkodTS L381R/K501R; **(G)** Tkod-Pol wild type. The primer-template used was: 5′-GGGGATCCTCTAGAGTCGACCTGCAGGGCAA 3′-CCCCTAGGAGATCTCAGCTGGACGTCCCGTTCGTTCGAACAGAGG. The primer was labeled at its 5′-terminus with fluorescein.

Primer-template extensions used the fluorescent-labeled primer-templates given in the legends to Figures 2, 3 and supplementary data Figures S2, S3. These reactions were conducted, at 30°C, in 400 μl of 20 mM Tris pH 8.0, 10 mM KCl, 10 mM (NH_4_)_2_SO_4_, 2 mM MgSO_4_, 0.1% (v/v) Triton X-100 and 40 μ g of bovine serum albumin. 400 μM of each dNTP was used with 10 nM primer-template and reactions were initiated by adding the polymerase to a final concentration of 500 nM. Subsequently 40 μl aliquots were sampled at appropriate time-points by quenching with an equal volume of stop buffer (95% formamide, 10 mM EDTA, 10 mM NaOH, 2 μM of a competitor oligodeoxynucleotide and 0.05% xylene cyanol indicator dye. Primer-templates were denatured by heating at 100°C for 10 min followed by cooling on ice. The competitor, which has the same sequence as the fully extended primer but lacks the fluorophore, prevents significant re-hybridization of the fluorescent products to the template (Russell et al., 2009). The samples (20 μ l) were resolved on a denaturing 17% polyacrylamide gel containing 8 M urea run at 4.5 Watts for 4.5 h and visualized using a Typhoon scanner with ImageQuant software (GE Healthcare). Exonuclease assays were performed similarly, save for omission of the dNTPs and data were fitted to a first order reaction (% substrate remaining = 100e^−kt^ + offset; *k* = rate constant, *t* = time) using GraFit (Erithacus Software, London, UK), giving rate constants.

### Real time PCR

Real-time PCR used a Rotor-Gene 6000 thermocycler (Corbett Research, Qiagen). Genomic DNA from *S. cerevesiae* was used as the template, with the DNA Pol 2 gene targeted for amplification. A common forward primer (TACGTACCGCCGCAATACAATGGCAGG) and four different reverse primers (TCGAATTGCCGCCGCCATTACTACCAC, TCGACTTGAAGCTCCCACCCTCTTCATC, GGCGTCAACTTTTTCCGAGCCATTTGC and TCATCGAACATGTCCAAGCCGTGAATCTTAC) were used to amplify lengths of 145, 232, 543, and 1040 base pairs. Reactions were carried out in 25 μ l that contained 30 ng *S. cerevesiae* genomic DNA (Novagen), 1 μM of each primer, 400 μM of each dNTP, and 2.5 μ l of SYBR green (10,000 × stock in dimethlysulfoxide, Invitrogen; initially diluted 1000-fold with water). The reactions were initiated by adding the polymerase (final concentration 20 nM) in the same buffer used for primer-template extensions. The PCR consisted of 1 × 95°C for 2 min followed by 40 cycles of: 95°C (10 s)/58°C (20 s)/72°C (the time used for the 72°C extension step varied as indicated in the results). On completion of the PCR a melt curve analysis, consisting of a 90 s pre-melt step at 67°C followed by a temperature increase to 95°C at 0.2°C per second, was carried out. 20 μl of the real time PCR mixtures were run on a 1% agarose gel (detection with ethidium bromide) in order to verify that amplification resulted in a product of the correct size.

### PCR

Amplification of a stretch of DNA ~5 kbases long within the plasmid pET17b[Pfu-Pol] (Evans et al., 2000) was carried out in 50 μ l using TCTGCTATGTGGCGCGGTATTATCC and CAACTCAGCTTCCTTTCGGGCTTTG (1 μ M each) as primers, 400 μ M of each of the 4 dNTPs and either 20 or 100 nM of polymerase. 50 ng of pET17b[Pfu-Pol] was used with two buffers, either 20 mM Tris-HCl pH 8 or 20 mM Bicine-NaOH pH 9 both containing 10 mM KCl, 10 mM (NH_4_)_2_SO_4_, 2 mM MgSO_4_, 0.1% (v/v) Triton-X100 and 5 μ g bovine serum albumin. The PCR cycle comprised: pre-heat at 98°C (2 min); 30 cycles of 95°C (30 s), 60°C (30 s), 70°C (5 min); final hold at 70°C for 5 min. 20 μ l of the PCR mixtures (expected size of the correct amplicon ~5 kb) were analyzed using 1% agarose gel electrophoresis with ethidium bromide staining.

### Polymerase fidelity

Fidelities were determined using pSJ2, an assay based on gap-filling of the single stranded *lacZ*α segment within the plasmid by a polymerase *in vitro*. The gapped derivative of pSJ2 (1 nM) was fully extended using the polymerase (100 nM) in 20 μ l of 20 mM Tris (pH 8.0), 10 mM KCl, 10 mM (NH_4_)_2_SO_4_, 2 mM MgCl_2_, 0.1% (v/v) Triton X-100, 2 μg bovine serum albumin and 250 μM of each of the four dNTPs. Extension was carried out at 70°C for 30 min and the mixture was used to transform *E. coli* Top 10 cells, which were plated on LB agar (containing X-gal, IPTG and ampicillin) and scored for blue/white colonies. Ratios of blue/white colonies were converted to mutation frequency and error rate as previously described (Jozwiakowski and Connolly, 2009; Keith et al., 2013).

### Primer-template binding

The binding of the polymerases to primer-templates was determined using fluorescence anisotropy with hexachlorofluorescein-labeled DNA (Shuttleworth et al., 2004). The buffer conditions are detailed in the supplementary data (Figure S6).

### Processivity

Four hundred μ l of 20 mM Tris pH 8.0, 10 mM KCl, 10 mM (NH_4_)_2_SO_4_, 1 mM EDTA, 0.1% Triton X-100, 40 μ g of bovine serum albumin, 400 μM of each of the four dNTPs, 40 nM primer-template (sequence given in the legend to Figure 7) and 500 nM of polymerase were pre-incubated at 50°C for 5 min. Reactions were initiated by the simultaneous addition of 3 mM MgSO_4_ and 10 μM of a uracil-rich oligodeoxynucleotide trap (sequence given in legend to Figure 7) and polymerization allowed to proceed at 50°C. Aliquots of 40 μ l were withdrawn at appropriate times and extension determined using denaturing gel electrophoresis as described above.

### DSF analysis

The thermal stabilities of the polymerases were analyzed by heating samples in a Rotor-Gene 6000 (Corbett Research, Qiagen) in the presence of SYPRO Orange and measuring any increase in fluorescence. Thermal melts were carried out in 100 μ l of 40 mM HEPES, pH 7, containing 400 mM NaCl, 2 M guanidinium hydrochloride, 10 μ l of SYPRO Orange (5000 × stock in dimethylsulfoxide, Sigma; initially diluted 100-fold with water). The polymerases were used at a concentration of 2 μ M. Excitation and emission were at 470 nm and 555 nm, respectively. The temperature increased from 25 to 100°C at a rate of 1°C per min. Data analysis was carried out with the Rotor-Gene 6000 series software and the melting profiles are presented as first derivatives.

## Results

### Manipulating amino acids and domains of Pfu-Pol to make it more similar to Tkod-Pol

TKod-Pol contains seven arginines at the forked-point, four of which are replaced in Pfu-Pol (Figures 1A,B). The absent arginines have been investigated by introducing them into Pfu-Pol, initially as the single amino acid substitutions, Pfu-PolM247R, T265R, and K502R. The situation at Tkod-Pol position 381 is more complex, due to the insertion of an additional leucine at position 381 in Pfu-Pol (Figure 1B). Therefore, two Pfu-Pol variants have been created, corresponding to the potential sequence line ups shown in Figure 1B. The deletion of L381, to give Pfu-PolL381Δ, removes the extra amino acid, which may bring R382 in Pfu-Pol into register with R381 in Tkod-Pol. Additionally the direct substitution mutation, Pfu-PolL381R, has been prepared, corresponding more closely to structural data (Figure 1C). Insertion of L381 also means that lysine 502 in Pfu-Pol corresponds to arginine 501 in TKod-Pol. Several of the single amino acid modifications to Pfu-Pol showed improved ability to copy DNA (next section). To generate incremental increases in polymerization activity, two double mutants, Pfu-PolM247R/L381R and Pfu-PolL381R/K502R and a triple variant, Pfu-PolM247R/L381R/K502R were prepared.

A second Pfu-Pol mutant has the thumb domain (amino acids T591 to S775 at the carboxyl terminus) replaced with the corresponding region from Tkod-Pol (named Pfu-TkodTS, TS = thumb swap). There are multiple differences in the amino acid sequences that comprise the thumb domains of the two polymerases (Figure 1D; supplementary data figure S1). Of the amino acids in the Tkod thumb that contact DNA directly, only two, R709 and G711 (shown in green in Figure 1D), are changed in Pfu-Pol, to proline and serine, respectively. However, many Tkod residues, immediately adjacent to a DNA-contacting amino acid, also vary in Pfu-Pol (supplementary data Figure S1). The numerous variations in sequence make it a sizeable task to probe the contribution of individual amino acids; rather a complete thumb transplant was used. Finally the most advantageous “forked-point” double mutant (L381R/K502R) has been combined with the thumb domain swap.

### Primer-template extension by DNA polymerases

**Figure 4:**
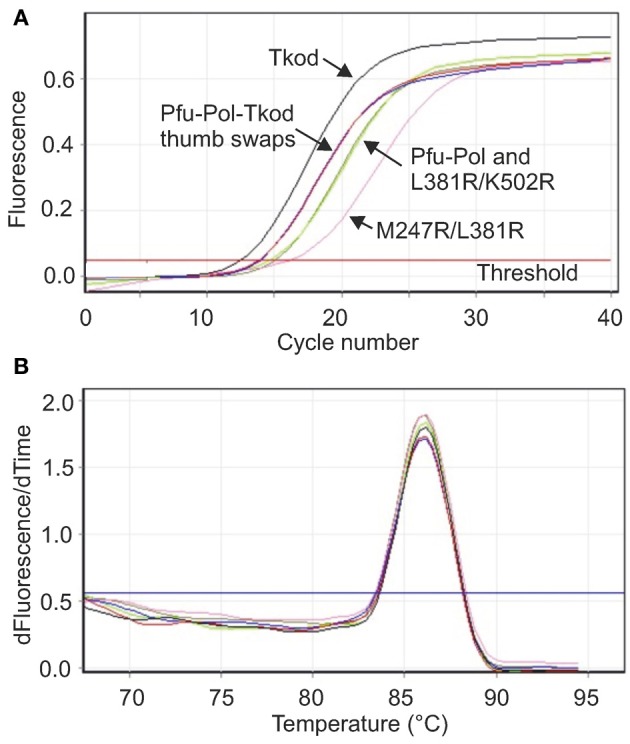
**Real time PCR analysis of polymerase performance. (A)** Amplification of a stretch of yeast genomic DNA 232 bases in length using 10 s extension. The lines that correspond to the individual polymerases are identified on the figure. Pfu-Pol TS and Pfu-Pol TS L381R/K502R (indicated Pfu-Tkod thumb swaps) gave near superimposable lines. Likewise the lines for Pfu-Pol and the double mutant L381R/K502R overlapped strongly. **(B)** Melting temperature analysis (first derivative showing the rate of change of temperature with time against time) of the amplicons generated in **(A)**. All the polymerases gave exclusively the desired product as indicated by a single peak with a *T*_*m*_ of 86°C. As all the lines are essentially identical the individual polymerases have not been identified. In both panels only a single line for each polymerase is shown but all experiments were carried out in triplicate.

**Figure 5:**
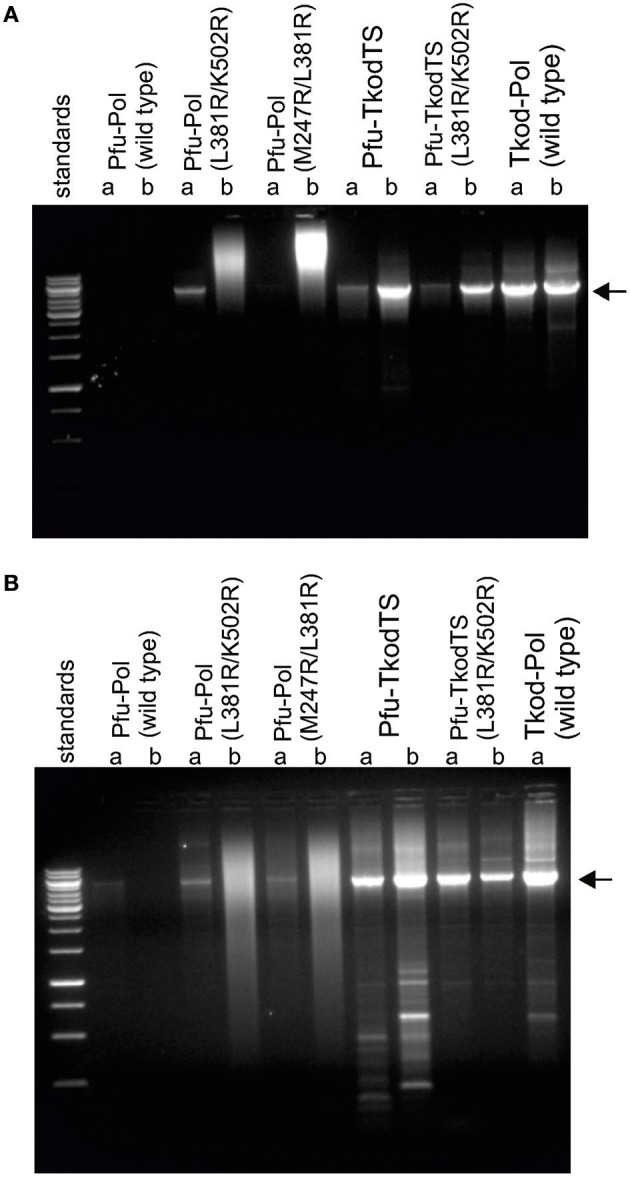
**PCR amplification of ~5 kb of DNA in pET17b[Pfu-Pol] by polymerase variants**. Reactions were carried out using the polymerase variants indicated (*a* = 20 nM; *b* = 100 nM) in two buffer systems (**A** = Tris-HCl pH 8; **B** = Bicine-NaOH pH 9). Analysis was by 1% agarose gel electrophoresis with ethidium bromide staining. The standards comprise a 1 kb ladder with the more intense bands at 1, 3, and 6 kB. The expected PCR product (~5 kb) is indicated with the arrow.

Polymerase activities were initially compared using several primer-templates, a simple extension assay useful for indicating the effectiveness with which DNA is copied. The single amino acid substitutions Pfu-Pol M247R and K502R generated full-length product more rapidly than wild type (supplementary data, Figure S2). At amino acid position 381, where two mutations were made, L381R appeared slightly better than L381Δ and both were superior to wild type. Pfu-Pol T265R was the only instance of a substitution giving poorer extension (supplementary data, Figure S3). Building on the single-swaps, two double mutants (M247R/L381R and L381R/K502R) were created; both extended the primer-template significantly more rapidly than wild type and slightly faster than their single parents. Faster extension with the double mutants was consistently observed with three primer-templates (Figures 2, 3; supplementary data Figure S2). As can be seen in Figure 2, wild type Pfu-Pol required 10–20 min before full length product became visible, whereas completely extended primer was apparent after 1–2 min with both of the doubles. An alternative primer-template (Figure 3) again indicated more rapid extension with the double mutants (compare the five and twenty minute time points for wild type, M247R/L381R and L381R/K502R in Figure 3). This figure additionally demonstrates that the triple mutation (M247R/L381R/K502R) shows little additive improvement. Although Pfu-Pol M247R/L381R and L381R/K502R are superior to wild type, they do not match the performance of Tkod-Pol, where full extension is apparent after less than 10 s (Figures 2, 3). The assay was also shows that Pfu-TkodTS extends primer-template more rapidly than wild type (Figure 3). With Pfu-TkodTS the reaction is almost complete after 5 min, whereas little full length product is seen with the wild type after this time. Even faster extension was seen when Pfu-TkodTS/L381R/K502R was investigated, with the reaction finishing in 1–2 min. Extensions were carried out at 30°C, away from the temperature optima of about 75°C for Pfu-Pol and Tkod-Pol (Takagi et al., 1997). Maybe, as temperature decreases, the activity of Pfu-Pol declines more steeply than Tkod-Pol, explaining the observed superior performance of Tkod-Pol at 30°C. However, Tkod-Pol maintained its advantage in both real time and standard PCR (Figures 4, 5 respectively), techniques that involve DNA synthesis near the temperature optimum. Based on primer-template extensions, four mutants, M247R/L381R, L381R/K502R, Pfu-TkodTS, and Pfu-TkodTSL381R/K502R were selected for more detailed investigation.

### Real time PCR analysis

A key motivation in manipulating Pfu-Pol was to produce superior PCR enzymes and, to allow direct identification of useful mutants, priority was given to PCR-based assays. Initially real time PCR (RT-PCR), commonly used to measure DNA and RNA levels (Saunders and Lee, 2013), was applied to investigate polymerase performance. During RT-PCR a *C*_*t*_ value defines the number of cycles taken for product to become apparent; the more effective the polymerase, therefore, the lower the *C*_*t*_. In these experiments, yeast genome DNA targets of 232, 543, and 1040 bases were generated and the products detected using the fluorophore SYBR green. With the shortest amplicon (232 bases), Pfu-Pol and the double mutant L381R/K502R generated product after a similar number of cycles (*C_t_* 15.30 and 14.93, respectively) and M247R/L381R was slightly slower (*C*_*t*_ 15.96). Here, the behavior of M247R/l381R is anomalous as it appeared superior to the wild type in every other test of polymerization ability. Tkod-Pol produced product more rapidly than the wild type i.e., had a lower *C*_*t*_, and the two thumb swap mutants showed intermediate *C*_*t*_ values. As an example the real time PCR data found with the short amplicon is shown in Figure 4 and all *C*_*t*_ values are summarized in Table 1. Further information was obtained using a longer amplicon of 543 bases and varying the extension time. The double mutants appeared superior to Pfu-Pol, either giving a product where none was produced by the parent or generating amplicons more quickly; L381R/K502R demonstrated better performance than M247A/L38R. Greater efficiency was again apparent for Tkod-Pol, which generated products more rapidly as measured by *C*_*t*_ values. Similarly the thumb swap mutants showed better performance than Pfu-Pol (wild type and the double mutants) but were inferior to Tkod-Pol (Table 1). Confirmation of these trends came from the use of the longest amplicon (1040 bases) with four different extension times. Overall, as summarized in Table 1, Tkod-Pol gives the best performance in RT-PCR followed, in order, by Pfu-TkodTSL381R/K502R, Pfu-TkodTS, Pfu-PolL381R/K502R, Pfu-PolM247R/L381R, with Pfu-Pol wild type, the worst enzyme. This ranking is an excellent match to the results seen in primer-template extensions. While RT-PCR is a straightforward method for characterizing polymerases, incorrect products are often produced. Therefore, the integrities of the 232, 543, and 1040 base products were checked using melting temperature analysis (all three had similar *T*_*m*_ values of between 86 and 88°C; for an example see Figure 4) and by gel electrophoresis to confirm the presence of product with the expected length (supplementary data, Figure S4). In Table 1, *C*_*t*_ values are only quote if the anticipated product comprised at least 95% of the amplification mixture.

**Table 1:** **RT-PCR performance of the polymerase variants**.

**Experimental conditions**		***C*_*t*_value of polymerase variant^b^**					
**Length of amplicon^a^**	**Extension time (s)**	**Pfu-Pol wild type**	**Pfu-Pol M247R/L381R**	**Pfu-Pol L381R/K502R**	**Pfu-TodTS**	**Pfu-TkodTS L381R/K502R**	**Tkod-Pol wild type**
232	10	15.30	15.96	14.93	13.90	14.07	12.58
543	10	NP	NP	NP	14.65	12.73	10.94
543	30	NP	12.20	10.55	8.61	8.07	6.72
543	60	11.78	11.07	10.04	8.60	8.04	6.52
543	90	11.16	10.46	9.66	8.54	7.80	6.54
1040	10	NP^c^	NP	NP	NP	NP	12.29
1040	30	NP	NP	NP	NP	16.90	11.05
1040	60	NP	NP	16.37	14.51	12.74	10.83
1040	90	NP	NP	13.85	12.66	11.10	10.75

a Yeast genome DNA was the target for amplification and primers (given in Materials and Methods) were selected to give the amplicon lengths indicated.

b The figures in the table represent the C_t_ value, the number of cycles required for the amplicon to become detectable. All experiments were conducted in triplicate with the C_t_ being the average of the three runs. In all cases were a figure is quoted, melting temperature analysis revealed a product with a T_*m*_ value of between 86 and 88°C and gel electrophoresis showed an amplicon of the correct length (Figure 4; supplementary data, Figure S4). In all cases the anticipated product comprised at least 95% of the total amplified material.

c NP, no product; either no product was produced or non-specific amplification occurred, giving either an incorrect product or a mixture of amplicons containing both the desired and non-specific products.

### PCR performance of polymerases

To determine if the more rapid primer-template extensions and lower *C*_*t*_ values exhibited by the Pfu-Pol mutants translated into improved standard PCR performance, amplifications of a 5 kb stretch of DNA were carried out. Two buffers with pH of 8 and 9 were used and the polymerases were tested at two concentrations, 20 or 100 nM. The results are given in Figure 5 and show that wild type Pfu-Pol failed to generate substantial amounts of product, traces being apparent only at pH 9 with 20 nM enzyme. The double mutants M247A/L381R were better; at 20 nM levels both gave obvious product at pH 9, L381R/K502R also yielded product at pH 8. However, at the higher concentration of 100 nM the doubles gave smeared bands, suggesting non-specific amplification. The thumb swaps showed marked PCR improvement, at both pH values and protein concentrations a clear and intense (apart from pH 8, 20 nM) product band was visible, suggesting efficient amplification. Some non-specific products were apparent, particularly at pH 9. Wild type Tkod-Pol also performed well, perhaps marginally better than the two thumb swap mutants. Non-specific smaller products were also observed with Tkod-Pol at pH 9. The PCR results concur with all those above; the double mutants are superior to wild type Pfu-Pol and the thumb swaps even better. In these experiments the thumb swaps approach the performance exhibited by wild type Tkod-Pol.

### Fidelity of DNA polymerases

**Figure 6:**
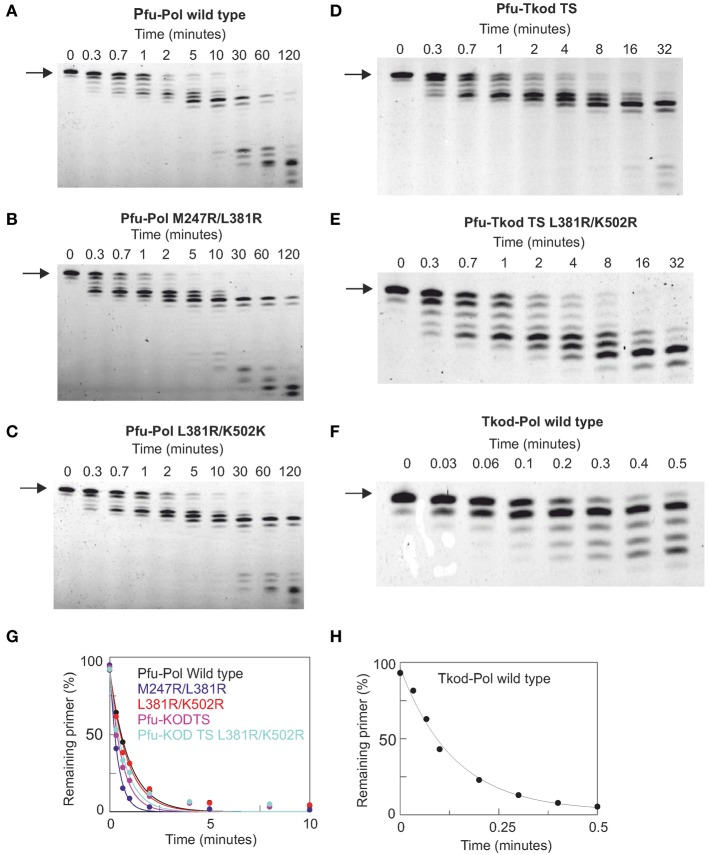
**Proof reading 3′-5′ exonuclease activity by archaeal family-B DNA polymerases. (A–F)** Gel electrophoresis analysis of primer strand degradation seen with a base mismatch primer-template using the polymerase variants indicated. The starting primer is shown arrowed. Panels **(G)** and **(H)** show fits of the data (as % primer remaining with time) to a single exponential decay to give the rate constants summarized in Table 2. The primer-template used was: 5′-GGGGATCCTCTAGAGTCGACCTGC 3′-CCCCTAGGAGATCTCAGCTGGACAACCGTTCGTTCGAACAGAGG. The primer was labeled at its 5′-terminus with fluorescein. The mismatched bases are shown underlined,

DNA polymerase accuracy is crucial for PCR and the main reason archaeal enzymes are widely applied. Two techniques were used to check the fidelity of the enzymes. Initially proof reading exonuclease activity was directly measured using a primer-template containing a base mismatch at the point of extension. A protein concentration (500 nM) in excess of primer-template (10 nM) was used and exonuclease activity observed by monitoring the removal of the mismatched base at the 3′-end of the primer. All Pfu-Pol mutants demonstrated slightly faster exonuclease rate constants than wild type, although increases were at most a factor of two (Figure 6, Table 2). Noticeably wild type Tkod-Pol showed more pronounced activity, about 7.5 times faster than Pfu-Pol. The plots shown in Figure 6 and the rate constants in Table 2 measure the disappearance of initial primer and so report on removal of the mismatched base. It is abundantly clear that subsequent degradation of the primer can occur, as a consequence of exonuclease activity at Watson-Crick base pairs. With Tkod-Pol accumulation of the n-1 product is clear, suggesting preferential degradation of mismatched bases. Equivalent accumulation is less obvious with Pfu-Pol, inferring less discrimination between mismatches and *bona fide* base-pairs in this case. To further investigate the proof reading activities of Pfu-Pol and Tkod-Pol were compared using a fully complementary primer-template. With these undamaged substrates the exonuclease activity of Tkod-Pol was only 1.5 fold faster than Pfu-Pol, less than the factor observed with the mismatched substrate (supplementary data, Figure S5). The faster exonuclease rate of Tkod-Pol, compared to Pfu-Pol, with mismatches, followed by near equal reactivity at normal bases accounts for the n-1 product accumulation seen in Figure 6. Exonuclease activities were greater with mismatched than fully complementary substrates (Table 1); an expected observation as mismatches are easier to unwind, a necessary step for proof reading (Reha-Krantz, 2010).

**Table 2:** **Summary of the properties of the polymerases investigated**.

**DNA-Pol**	***K*_*D*_ (nM)^a^**	***k*_exo_ (min^−1^) (mis-paired DNA)^b^**	***k*_exo_ (min^−1^) (base-paired DNA)^c^**	**Processivity^d^**	**Melting temperature (°C)^e^**
Pfu-Pol wild type	251 ± 23	1.0 ± 0.1	0.17 ± 0.02	1	93.2 ± 0.5
Pfu-Pol M247R/L381R	70 ± 9	2.2 ± 0.1	nd	1	95.7 ± 0.2
Pfu-Pol L381R/K502R	107 ± 13	1.2 ± 0.1	nd	1–3	94.5 ± 0.8
Pfu-TkodTS	95 ± 7	1.8 ± 0.1	nd	1–3	95.3 ± 1.0
Pfu-TkodTS L381R/K502R	50 ± 7	1.7 ± 0.1	nd	3	95.3 ± 0.6
Tkod-Pol wild type	276 ± 18	7.6 ± 0.2	0.24 ± 0.01	3	82.9 ± 0.3

a The affinity for DNA measured using fluorescence anisotropy titrations (supplementary data, Figure S6). Each value is the average of three determinations ± the standard deviation. The primer-template used had the following sequence (Hex = hexachlorofluorescein): 5 '-HexGGGGATCCTCTAGAGTCGACCTGC 3 '-CCCCTAGGAGATCTCAGCTGGACGACCGTTCGTTCGAACAGAGG. (the mismatched bases are underlined.)

b Rate observed for the degradation of a mis-paired primer-template, determined with polymerase in excess of DNA (Figure 6). Each value is the average of three determinations ± the standard deviation. For the mis-paired DNA, the same sequence as given above was used with the underlined dG changed to dA, giving a dC:dA mis-match at the primer-template junction. Fluorescein (Flu) was used as indicator dye.

c Rate observed for the degradation of a fully base-paired primer-template, determined with polymerase in excess of DNA (supplementary data, Figure S5). Each value is the average of three determinations ± the standard deviation. This primer-template has the exact sequence given above and fluorescein (Flu) was used as indicator dye.

d Processity (number of dNTPs incorporated per binding event) of the polymerases measured using a uracil-containing single stranded DNA trap (Figure 7).

e Melting temperatures were determined by DSF in the presence of 2 M guanidinium hydrochloride (Figure 8). The T_*m*_ of the first transition observed is given as an average ± standard deviation from three measurements.

To complement the measurements of exonuclease activities, fidelities have been determined using a *lacZ*α plasmid-based assay (Jozwiakowski and Connolly, 2009; Keith et al., 2013). A gapped plasmid, which contains the *lacZ*α gene in the single stranded region, is fully extended, *in vitro* at 70°C, by a polymerase and then used for transformation of an appropriate *E. coli* strain. The observed ratio of white to blue colonies on plates contain the indicator X-gal, reflects the fidelity of the polymerase. For these experiments pSJ2 was used, a plasmid that has been characterized sufficiently to allow conversion of white/blue ratio i.e., the mutation frequency into error rate, the number of mistakes made per base incorporated (Keith et al., 2013). The results observed are given in Table 3, which demonstrates near identical error rates for each of the polymerases. All the mutants maintain the high accuracy associated with wild type Pfu-Pol and Tkod-Pol. Although Tkod-Pol has a measurably higher exonuclease activity than all the Pfu-Pol derivatives (Table 2), this does not translate into higher accuracy as determined using pSJ2. Fidelity critically depends on the balance between exonuclease and polymerase rates and with Tkod-Pol both are elevated compared with Pfu-Pol. The ratio of polymerase/exonuclease activities may be similar for both enzymes, leading to equal propensity to continue synthesis vs. engaging proof-reading and conferring similar overall fidelity on both proteins.

**Table 3:** **Fidelities of DNA polymerases determined using the *lacZ*α indicator pSJ2**.

**DNA-Pol**	**Total colonies^a^**	**Mutant (white) colonies**	**Mutation frequency (corrected)^b^**	**Error rate^c^**
Pfu-Pol wild type^d^	25,700	11	3.2 × 10^−4^	1.6 × 10^−6^
Pfu-Pol M247R/L381R	20,555	8	2.8 × 10^−4^	1.4 × 10^−6^
Pfu-Pol L381R/K502R	26,675	9	2.3 × 10^−4^	1.2 × 10^−6^
Pfu-TkodTS	39,814	16	2.9 × 10^−4^	1.5 × 10^−6^
Pfu-TkodTS L381R/K502R	31,704	12	2.7 × 10^−4^	1.3 × 10^−6^
Tkod-Pol wild type	28,028	11	2.8 × 10^−4^	1.4 × 10^−6^

a Sum of three independent experiments, each consisting of five repeats.

b The Mutation frequency is the ratio mutant (white) colonies/total colonies and has been corrected by subtracting the background mutation frequency of 1.1 × 10^−4^ found for gapped pSJ2 (Keith et al., 2013).

c The Error rate is the number of mistakes made by the polymerase per base incorporated. The determination of the error rate from the mutation frequency has been described previously (Keith et al., 2013).

d Data taken from an earlier publication (Keith et al., 2013).

### Primer-template binding

The influence of the mutations on primer-template binding was determined using fluorescence anisotropy with hexachlorofluorescein-labeled primer-templates. Previous studies, carried out at pH 7.5 and 100 mM NaCl (ionic strength = 100), indicated relatively poor binding of Pfu-Pol to primer-templates, a *K*_*D*_ of 270 nM being measured (Shuttleworth et al., 2004). As expected, binding affinity was increased at the lower salt concentration of 20 mM KCl (ionic strength = 20), reflected in a *K*_*D*_ of 32 nM (Richardson et al., 2013). In this publication binding titrations were performed in the same buffer used for extensions; pH 8.5 containing 10 mM KCl and 10 mM (NH_4_)_2_SO_4_ (ionic strength of 40). Under these conditions wild type Pfu-Pol bound the primer-template with a *K*_*D*_ of 251 nM (supplementary data, Figure S6). Tkod-Pol showed similar affinity (*K*_*D*_ = 276 nM) and so the more rapid primer-template extensions seen with this enzyme, cannot simply be accounted for by tighter binding to the DNA substrate. The two double mutants showed better interaction with DNA, by a factor a little greater than two for L381R/K502R and just over three for M247R/L381R (supplementary data, Figure S6). Surprisingly Pfu-KodTS bound primer-template (*K*_*D*_ = 95 nM) more strongly than either of the parent polymerases from which it is derived and a further improvement was observed with the Pfu-KodTS L381R/K502R (*K*_*D*_ = 50 nM). All The *K*_*D*_ values seen are summarized in Table 2.

### Processivity of DNA polymerases

The processivity of a polymerase is the number of dNTPs incorporated per binding event (Von Hippel et al., 1994; Bambara et al., 1995).For accurate measurement each primer should undergo only one round of extension and, under these single hit conditions, the number of dNTPs incorporated equals the processivity. Single hits can be achieved using a low concentration of polymerase, relative to primer-template, such that the probability of secondary initiation is low. Alternatively a trap, such as heparin or poly(dA-dT), can be used to sequester the polymerase after it dissociates from the primer-template (Bambara et al., 1995). Archaeal DNA polymerases bind tightly to uracil-containing DNA (Shuttleworth et al., 2004), enabling a 23-mer containing 5 uracil residues (Figure 7) to be used as trap. The uracil-rich trap bound all polymerase variants with *K*_*D*_ values in the 1–10 nM range (data not shown). For processivity determination 40 nM primer-template (sequence given in the legend to Figure 7) and 500 nM polymerase were pre-incubated in the absence of Mg^2+^ and reactions initiated by the simultaneous addition of the metal and 10 μ M of the trap. These stringent conditions, a 250-fold excess of a uracil-rich oligodeoxynucleotide with a high affinity for polymerase, should ensure efficient polymerase sequestration and inhibit re-binding to primer-template.

**Figure 7:**
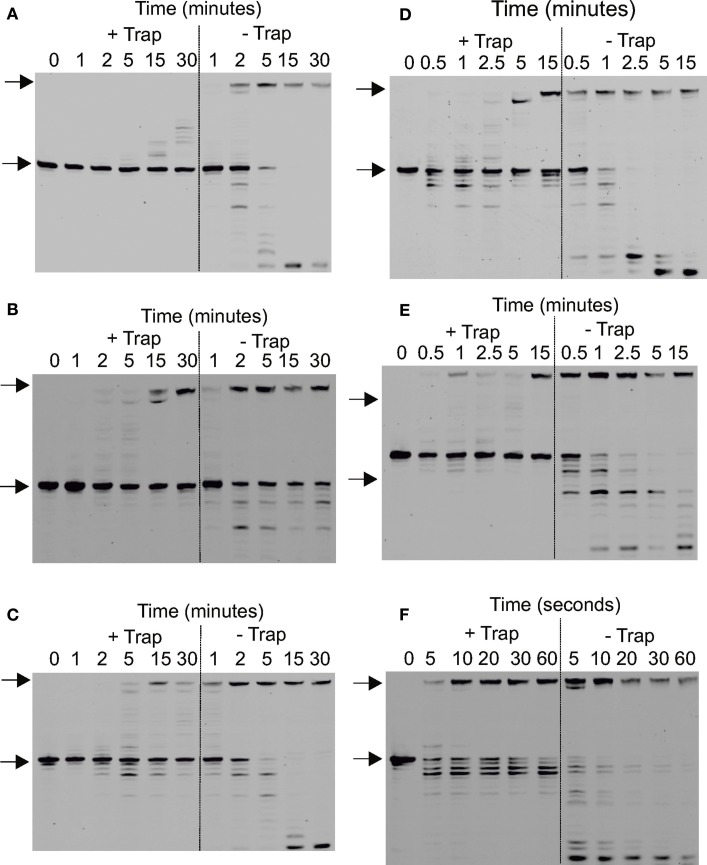
**Determination of the processivity of archaeal DNA polymerases. (A–F)** Gel electrophoresis analysis of primer strand extension seen using the polymerase variants and times indicated for: **(A)** Pfu-Pol wild type; **(B)** Pfu-Pol M247/L381; **(C)** Pfu-Pol L381R/K501R; **(D)** Pfu-TkodTS; **(E)** Pfu-TkodTS L381R/K501R; **(F)** Tkod-Pol wild type. The starting primer and fully extended product are both arrowed. The primer-template used was: 5′-GGGGATCCTCTAGAGTCGACCTGC 3′-CCCCTAGGAGATCTCAGCTGGACGACCGTTCGTTCGAACAGAGTACCTGGCTAT The primer was labeled at the 5′-teminus with fluorescein and the reaction initiated by the simultaneous addition of Mg^2+^ and the uracil rich single-stranded trapping oligodeoxynucleotide 5′-GTTGGUACUCTUAGUCTUTAGGT (extensions labeled + trap). For the extensions labeled—trap the competitor was omitted. Larger versions of each of the gel are given in the supplementary section (supplementary data Figure S7).

All polymerases were observed to have very low processivities at 250-fold trap excess and nearly identical results were see with a 100-fold excess (data not shown). Pfu-Pol acted largely in a distributive manner, with little product seen at short time intervals (Figure 7A; larger versions of all processivity gels are given in the supplementary data, Figure S7). A very faint band representing a single dNTP addition is seen after 2 min and +1 and +2 bands are visible after 5 min. Longer products were observed at 15 and 30 min but may result from multiple binding events. Thus the uracil trap is not perfect and at longer times polymerase “escape” is seen, maybe arising from trap degradation by the exonuclease activity. As expected, polymerization was more extensive when the trap was omitted (Figure 7A). Adding the trap in the pre-incubation step results in the abolition of the barely extended bands seen at short times; instead only slower mobility bands are observed after prolonged incubation, which correspond well with the similar bands observed when the uracil trap is used in the standard manner. Pfu-Pol has a processivity of 1 (values are summarized in Table 2), implying dissociation prior to adding even a single dNTP is highly likely. With Tkod-Pol a prominent band, corresponding to the incorporation of 3 dNTPs is seen at the shortest time (5 s) in the presence of the trap, representing the processivity (Figure 7E, Table 2). Tkod-Pol is more active than Pfu-Pol and even in the presence of trap full length product, presumably due to multiple binding events, was seen at short times. As expected these bands were more prominent when the trap was left out. Pronounced exonuclease activity, evidenced by shortened primer products was also apparent. The four mutants showed processivity profiles near those of the wild types (Figures 7B–D, Table 2). The double mutant M247A/L381R was very similar to Pfu-Pol with a faint band, representing incorporation of a single dNTP just visible at the one minute time point. L381R/K502R appeared marginally better, the band indicating one dNTP addition was predominant at times of 1 and 2 min, but fainter bands corresponding to the +2 and +3 products were seen. The processivity of this mutant, 1–3, may be slightly improved. Processivity does not need to be a single integral value; rather polymerases show a spread of dNTP incorporations per binding event. Pfu-Kod(TS) also demonstrated a processivity of between 1 and 3 (Figure 7C). Finally Pfu-TkodTSL381R/K502R appeared to be equivalent to Tkod-Pol with a single band at the +3 location visible at short times. Overall Tkod-Pol has higher processivity than Pfu-Pol (3 vs. 1) and with the mutations values are shifted toward Tkod-Pol.

The processivities determined here, using a uracil-rich oligodeoxynucleotide trap, are much lower than previously observed. Values of 270 and >300 have been reported for Tkod-Pol and of 6, <20 and 80 for Pfu-Pol (Takagi et al., 1997; Wang et al., 2004; Kim et al., 2007). None of these studies used a trap to hinder re-binding of the polymerase and secondary extension and the change in protocol may explain the discrepancy. All studies agree, however, that Tkod-Pol is more processive than Pfu-Pol. Yeast DNA polymerase δ, similar in structure to the archaeal enzymes (Swan et al., 2009), shows a processivity of 2–3 when evaluated using a trap (Hogg et al., 2014).

### Thermostability of DNA polymerases

**Figure 8:**
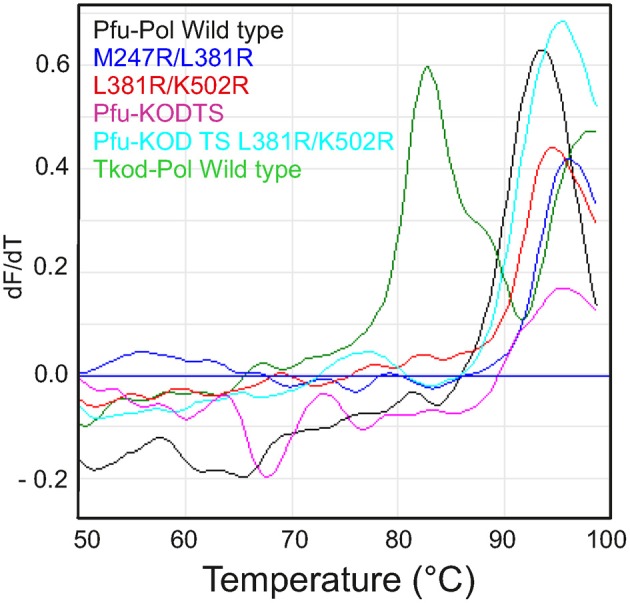
**Differential scanning fluorimetry (DSF) profiles describing the thermal unfolding of the polymerases**. The first derivatives of the DSF profiles are shown with *dF*/*dT* indicating the change in fluorescence (relative units). Individual polymerases are identified by the color coding given in the figures.

The ability to survive elevated temperature is an essential polymerase feature for the PCR. Both archaea, from which the polymerases used in this study are isolated, are hyper-thermophiles, but their preferred growth temperatures differ. *Pyrococcus furiosus* grows optimally at 100°C and can survive at 110°C, whereas *Thermococcus kodakarensis* grows best at 85°C and can tolerate 94°C (Fiala and Stetter, 1986; Borges et al., 2010). Our group earlier measured the thermostability of Pfu-Pol using differential scanning fluorimetry (DSF), where the protein is subject to a steady increase in temperature in the presence of the dye SYPRO orange (Killelea and Connolly, 2011). As thermally-induced unfolding takes place the dye binds to exposed hydrophobic regions, resulting in an increase in fluorescence that can be measured using a real time PCR apparatus. Pfu-Pol was found to be extremely thermostable with the unfolding transition being incomplete at 100°C, the maximum achievable with this technique. Addition of guanidinium hydrochloride (GuHCl) destabilizes Pfu-Pol, making thermal unfolding more accessible. A Pfu-Pol mutant, that lacked two disulphide bridges and was more heat sensitive, demonstrated two well separated melting transitions. Most likely the wild type behaves similarly but the second transition cannot be observed as it takes place above 100°C (Killelea and Connolly, 2011). In the present study 2M GuHCl was used to bring heat-induced unfolding into the DSF range and the melting profiles are shown in Figure 8. Tkod-Pol shows two melting transitions, the first of which has a *T*_*m*_ of 82.9°C (all *T_m_* values are summarized in Table 2). Pfu-Pol is more stable with a *T*_*m*_ of 93.2°C. As postulated earlier it is probable that a second melting event for Pfu-Pol takes place above 100°C; therefore, only the first observed transitions have been used for comparing unfolding. The *T*_*m*_ of 93.2°C is slightly higher than that of 89.7°C observed earlier for Pfu-Pol in 2 M GuHCl (Killelea and Connolly, 2011). Such variation may be accounted for by the different NaCl concentrations used, 400 mM here, 200 mM previously. Unsurprisingly the double mutants of Pfu-Pol, M247R/L381R (*T*_*m*_ = 95.7°C and L381R/K502R (*T*_*m*_ = 94.5°C) retain the thermostability of the wild type, if anything being slightly more heat resistant. More unexpectedly the thumb swaps, in which about 24% of the protein is derived from Tkod-Pol, fully retain the stability of Pfu-Pol, with no lowering of *T*_*m*_, toward that of Tkod-Pol is (Figure 8 and Table 2).

## Discussion

The use of the PCR across biological, medical, veterinary, agricultural and forensic sciences has aroused considerable interest in thermostable DNA polymerases and many enzymes, especially from the *Thermococcales* order of the archaea, are employed (Terpe, 2013). Tkod-Pol and Pfu-Pol are the most established and several investigations have pointed out the advantageous nature of the former in terms of speed and processivity (Takagi et al., 1997; Nishioka et al., 2001; Kim et al., 2007). The abundance of arginines at the “forked-point” has been offered as one reason for the high processivity of Tkod-Pol (Hashimoto et al., 2001; Kim et al., 2008), but the absence of a closed ternary complex (enzyme/primer-template/dNTP) limits knowledge on the exact functions of these amino acids. It was originally proposed that Tkod-Pol R247 may separate the primer-template and stabilize the denatured substrate, in a similar manner to corresponding amino acid in the bacteriophage RB69 polymerase (Shamoo and Steitz, 1999; Hashimoto et al., 2001). More recent studies have suggested that this amino acid is relatively unimportant (Aller et al., 2011; Richardson et al., 2013). The thumb domain of archaeal family-B polymerases, and indeed all DNA polymerases, is responsible for binding double-stranded DNA and expected to be critical in DNA translocation and processivity. Several “forked-point” arginines are missing in Pfu-Pol and the thumb domain shows subtle differences between the two enzymes. A major aim of this study was to graft these elements into Pfu-Pol in the hope of creating better PCR enzymes.

As “forked-point” arginines and/or the thumb domain of Tkod-Pol are introduced into Pfu-Pol, extension rates become faster, PCR performance improves and processivity, albeit only from one to three, is increased. In all cases the superior thermostability of Pfu-Pol is retained and there is no decrease in fidelity. The influence appears cumulative with the best mutant, combining both “forked-point” and thumb alterations. Pfu-TkodTS L381R/K502R represents an end-point (the entire thumb has been exchanged and addition of further missing arginines e.g., R246 does not enhance properties), yet still falls short of the performance of Tkod-Pol. This suggests other regions of the polymerase may play a role and these may subtly differ between Tkod-Pol and Pfu-Pol. One important area is the fingers domain, made up of two long α-helices (Hopfner et al., 1999; Chapin-Rodriguez et al., 2000; Hashimoto et al., 2001; Kim et al., 2008), which undergoes a conformational change following dNTP binding to the polymerase/primer-template complex, to produce a catalytically competent “closed” ternary complex. The fingers domain is highly conserved in all DNA polymerases and plays a critical role in dNTP selection and accurate insertion of the incoming base into the extending primer (Brautigam and Steitz, 1998). The amino acid sequences of the fingers domain of Tkod-Pol and Pfu-Pol are similar, but not identical (supplementary data, Figure S1). Given the critical function of this area in catalysis, the small variations may impinge on PCR performance. A second key element may be the Y-GG/A motif (Brautigam and Steitz, 1998), shown to be important in the processivity, fidelity and PCR capability of archaeal polymerases (Bohlke et al., 2000). The sequences near this region are shown in Figure 1, corresponding to the amino acids near arginine 381 (Y-GG/A = YEGG for Tkod-Pol and YTGG for Pfu-Pol). The presence of an additional leucine (L381) in Pfu-Pol alters the location of the conserved tyrosine and glycines, moving them away from the DNA phosphate backbone and disrupting conserved protein-DNA interactions (Kim et al., 2008). Deletion of this leucine did result in slightly improved primer-template extension, but it is not clear if this mutation correctly lines up the subsequent arginine in Pfu-Pol with R381 of Tgo-Pol (Figure 1) and the L381R substitution was slightly superior (supplementary data, Figure S2). Of course Pfu-PolL381R still retains an extra amino acid and this may disrupt the downstream Y-GG/A motif as seen for the wild type. Finally communication between the exonuclease and thumb domains has been implicated in the co-ordination of polymerase and proof-reading exonuclease activities (Kuroita et al., 2005; Kim et al., 2008). Trans-domain interactions between amino acids in these regions appear to control the relative motions of the two domains, influencing catalytic activities. The inter-domain contacts appear to vary slightly between Tkod-Pol and Pfu-Pol, potentially impinging on PCR properties. Such considerations may be especially significant with the thumb swap mutants.

The significance of this publication is the demonstration that it is relatively simple to improve the PCR performance of Pfu-Pol by substituting individual amino acids and even an entire domain found in the closely related Tkod-Pol. The Pfu-Pol framework seems remarkably tolerant to substantial alteration, maintaining thermostability and fidelity. Future possibilities include incorporating elements from other *Thermococcales* family-B polymerases; a reasonable number are known (Figure 1) and many have been applied in the PCR (Terpe, 2013). Further improvement may be achieved by combining gene segments using random techniques such as DNA shuffling (Stemmer, 1994) or staggered extension process (StEP) (Zhao et al., 1998) and selecting for improved PCR performance with compartmentalized self-replication (CSR) (Ghadessy and Holliger, 2007). This approach may even be extending to environmental DNA from uncharacterized archaea, widening the genetic resource (Matsukawa et al., 2009). Experiments could target the “forked-point” and thumb, as well as other elements, suggested above to play a role in PCR ability. Such an undertaking should yield important clues about features of archaeal DNA polymerases that are important for efficient and robust PCR and may, ultimately, lead to superior reagents. It may be possible to develop DNA polymerases more suited to demanding PCR applications such as amplifying DNA from single cells or old/damaged/degraded sources and in the steps needed prior to high throughput DNA sequencing.

## Author contributions

All authors carried out experiments to acquire data and participated in analysing and interpreting the results. Bernard A. Connolly originally conceived the project and Ashraf M. Elshawadfy, Brian J. Keith, and Thomas Kinsman contributed to experimental design. Bernard A. Connolly wrote the first draft of the work and all authors contributed in critical revision. All authors concur with the final version and agree to take responsibility for the work and conclusions described within.

### Conflict of interest statement

Brian J. Keith was partially funded by Bioline, a company with commercial interests in PCR enzymes. Apart from this the authors declare that the research was conducted in the absence of any commercial or financial relationships that could be construed as a potential conflict of interest.
