# Supplementary material for: DNA polymerase hybrids derived from the family-B enzymes of Pyrococcus furiosus and Thermococcus kodakarensis: improving performance in the polymerase chain reaction
Source: Front Microbiol. 2014 May 27;5:224. doi: 10.3389/fmicb.2014.00224 (PMC4034419; doi:10.3389/fmicb.2014.00224)

Supplementary, figure S1: Line up of the amino acids in the thumb domains of Tkod-Pol and Pfu-Pol

```
TKod 590: tkkkyavideegkittrgleivrrdwseiaketqarvleallkdgdvekavrivkevteklsk
Pfu  591: tkkryavideegkvitrgleivrrdwseiaketqarvletilkhgdveeavrivkeviqklan

                                                                 * *
TKod 653: yevppeklviheqitrdlkdykatgphvavakrlaargvkirpgtvisyivlkgsgrigdrai
Pfu  654: yeippeklaiyeqitrplheykaigphvavakklaakgvkikpgmvigyivlrgdgpisnrai

Tkod 716: pfdefdptkhkydaeyyienqvlpaverilrafgyrkedlryqktrqvglsawlkpkgt
Pfu  717: laeeydpkkhkydaeyyienqvlpavlrilegfgyrkedlryqktrqvgltswlnikks
```

The amino acids in the thumb domain in Tkod-Pol that interact directly with DNA are shown in red (in two instances in green, also marked *). These interactions are from a Tkod-Pol/primer-template complex with the DNA bound in the polymerisation mode (pdb 4K8Z).[1] Amino acids that differ between Tkod-Pol and Pfu-Pol are highlighted as coloured pairs. Most are shown in blue, indicating that while the residues vary between the two proteins, the amino acid present in Tkod-Pol does not make a direct contact with DNA. Only in two instances, R709/P710 and G711/S712, (shown in green and additionally marked * are amino acids that contact the DNA in the Tkod structure, changed in Pfu-Pol.

[1]Bergen, K., Betz, W., Welte, W., Diederichs, K. & Marx, A. (2013) Structures of KOD and 9°N DNA polymerases complexed with primer template duplex. *ChemBiochem*. **14**, 1058-1062.

Supplementary, figure S1: Line up of the amino acids in the fingers domains of Tkod-Pol and Pfu-Pol

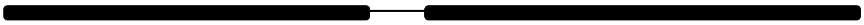

```
Pfu-Pol:  gfipsllghlleerqkiktkmketqdpiekilldyrqkaikllansfygyygy
Tkod-Pol: gfipsllgdlleerqkvkkkmkatidpiekklldyrqraikilansfygyygy
```

The fingers domain consists of two α-helices separated by a short loop. Amino acids that differ between Pfu-Pol and Tkod-Pol are highlighted in blue.

Supplementary figure S2: Primer-template extensions by “forked-point” mutations

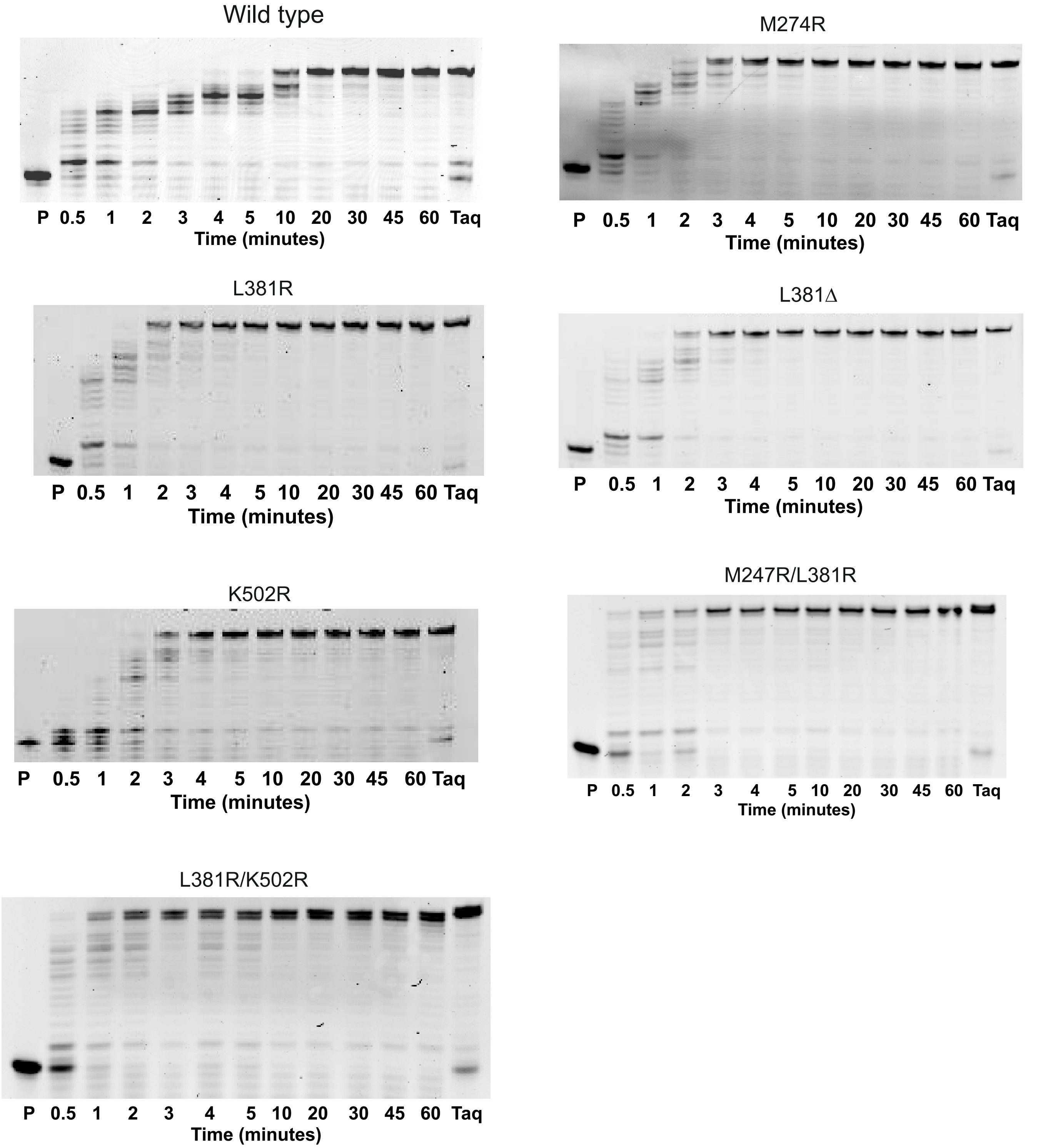

Initial primer-template extension experiments with Pfu-Pol “forked-point” mutations. Reactions were performed in the standard “extension buffer” (given in main paper) with 10 nM of primer-template (sequence given below; this is different to the primer-template made use of in the main paper), 200 nM Pfu-Pol (wild type and the mutants given above each gel) and 400 μM of each of the four dNTPs. The reactions were carried out at 30 °C for the times shown and analysed by gel electrophoresis. P = starting primer; Taq = reaction carried out with Taq-Pol for sixty minutes.

Primer-template:

5'-HexACCCGCGGGATATCGGCCCTTT-3'
3'   TGGGCGCCCTATAGCCGGGAAATCCGTTCGTCCGAACAGAGGTAT-5'

(Hex = hexachlorofluorescein)

Supplementary figure S3: Primer-template extension by “forked-point” mutation T265R

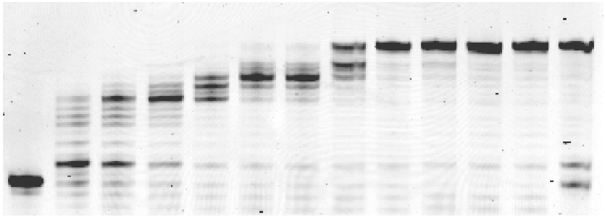

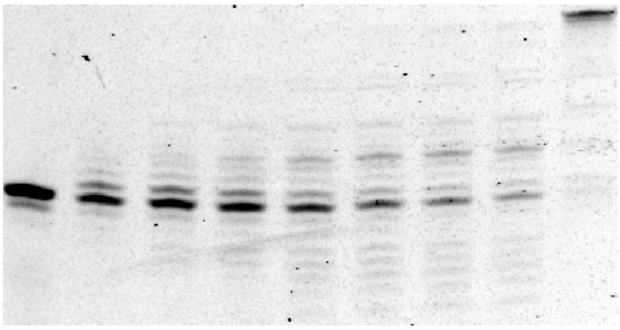

Primer-template extension experiments with Pfu-Pol “forked-point” mutation T265R. Reactions were performed in the standard “extension buffer” (given in main paper) with 10 nM of primer-template (sequence given below; this is identical to the primer-template made use of in the main paper), 200 nM Pfu-Pol (wild type and T265R) and 400 μM of each of the four dNTPs. The reactions were carried out at 30 °C for the times shown and analysed by gel electrophoresis. P = starting primer; Taq = reaction carried out with Taq-Pol for sixty minutes.

Primer-template:

5'-Cy5GGGGATCCTCTAGAGTCGACCTGC-3'
3'   CCCCTAGGAGATCTCAGGTGGACGACCGTTCGTTCGAACAGAGG-5'

(Cy5 = cyanine 5)

Supplementary figure S4: Agarose gel electrophoresis analysis of replicons produced by qPCR

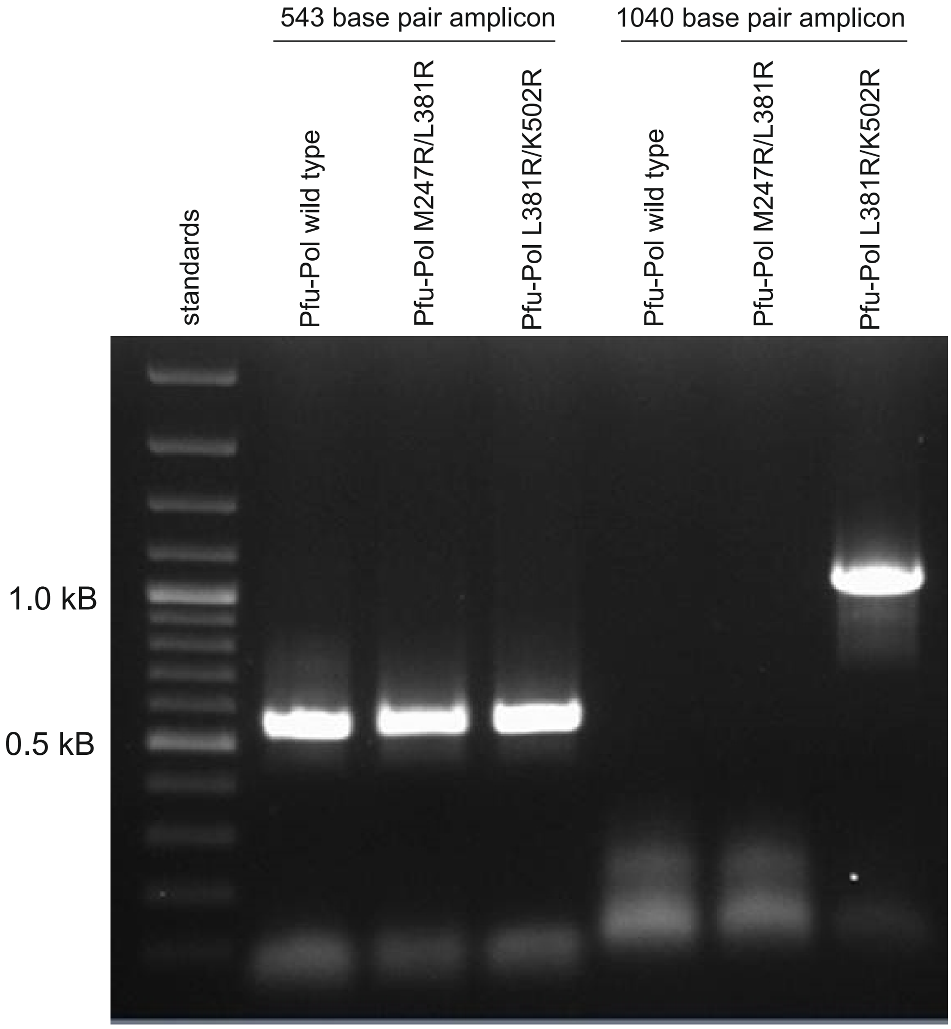

An example of the analysis of the amplicons produced during qPCR using agarose gel electrophoresis followed by staining with ethidium bromide. The data shown come from the amplification of DNA 543 and 1040 bases in length for 60 seconds using the polymerase variants shown above the gel lanes (see table 1, main paper). As can be seen all three enzymes gave the correct product with the 543 base DNA, whereas only Pfu-Pol L381R/K502R gave specific product with the 1040 base DNA. In all cases of successful amplification (see table 1, main paper) agarose gel electrophoresis indicated specific product formation.

Supplementary figure S5: Exonuclease activity of wild type Pfu-Pol and Tkod-Pol with a fully base-paired primer-template,

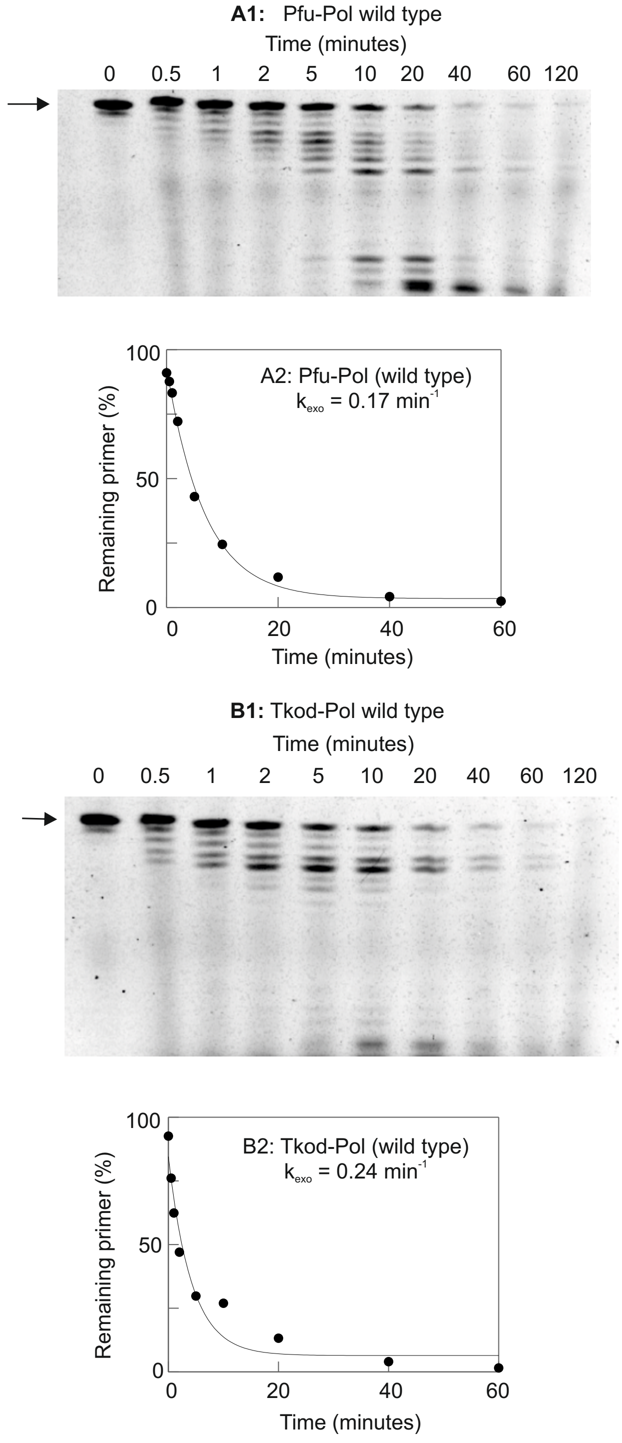

Proof reading 3'-5' exonuclease activity of wild type Pfu-Pol and Tkod-Pol with a fully base paired primer-template. A1: Gel electrophresis analysis of primer strand (indicated with error) degradation by Pfu-Pol. A2: Fit of the data (as % primer remaining with time) in A1 to a single exponential to give the rate constant. B1 and B2: As for A1 and A2 with Tkod-Pol. The sequece of the primer-tempalte used was:

5'- FluGGGGATCCTCTAGAGTCGACCTGC
3'- CCCCTAGGAGATCTCAGCTGGACGACCGTTCGTTCGAACAGAGG

Supplementary figure S6: Binding of archaeal family-B DNA polymerases to primer-templates

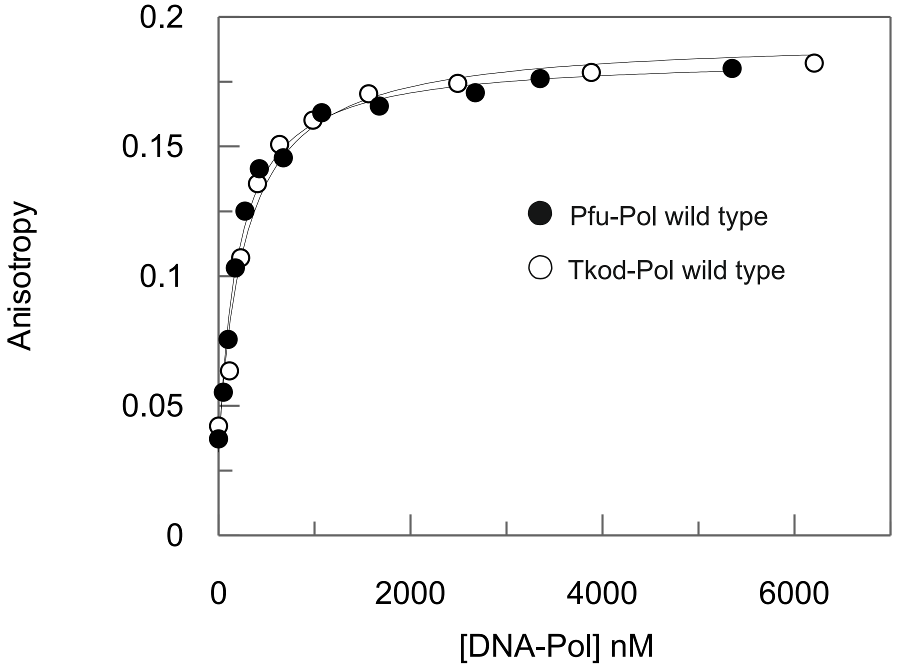

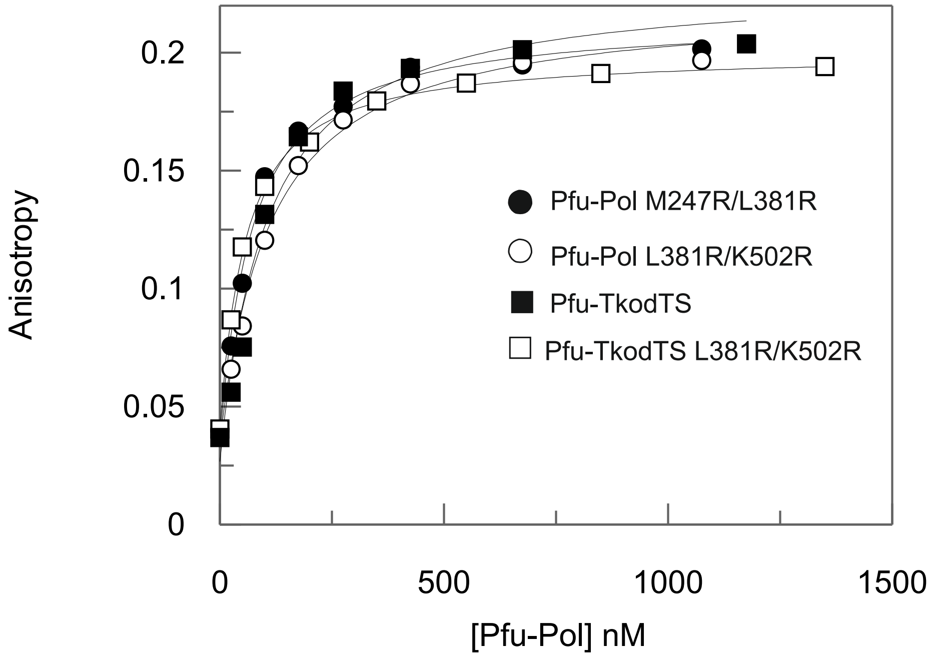

Binding of DNA polymerases to a primer-template (10 nM) measured in 20 mM Tris-HCl, pH 8.5, containing10 mM KCl, 10 mM $(NH_4)_2SO_4$ and 0.1 mg acetylated bovine serum albumin. Determination of binding constants relied on fluorescence anisotropy as previously described,[27] using the following primer-template (Hex = hexachlorofluorescein):

5'-HexGGGGATCCTCTAGAGTCGACCTGC
3'   CCCCTAGGAGATCTCAGCTGGACGACCGTTCGTTCGAACAGAGG

Supplementary figure S7: Larger versions of gels shown in figure 7 in main text

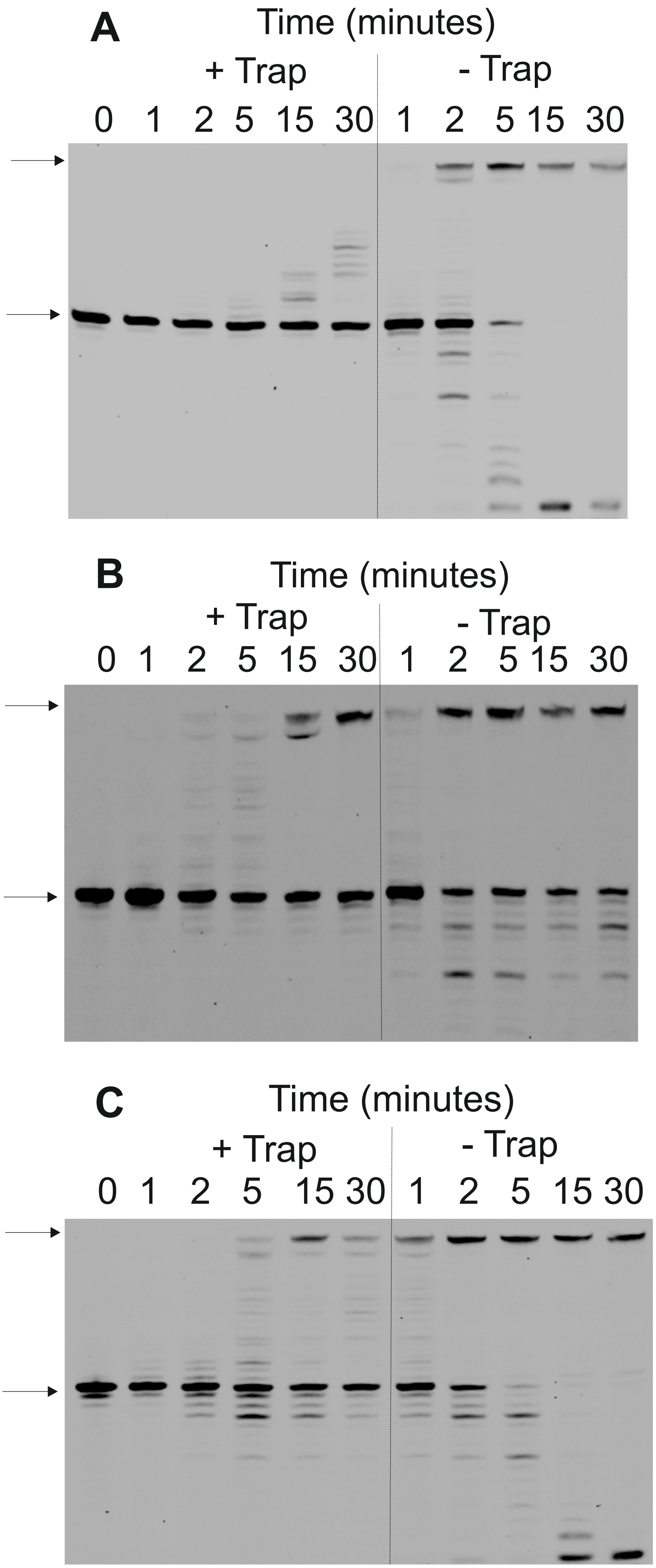

Supplementary figure S7: Larger versions of gels shown in figure 7 in main text

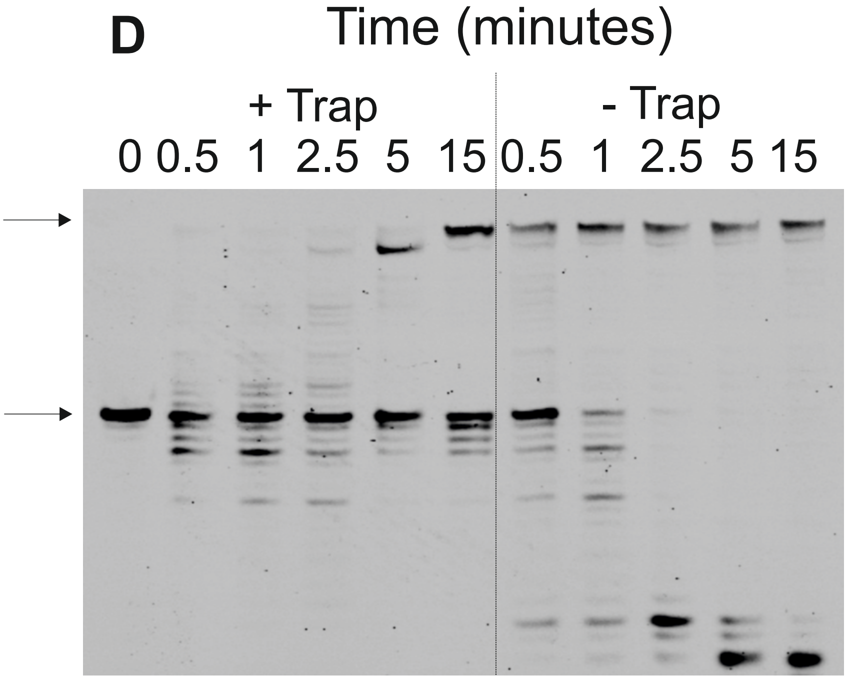

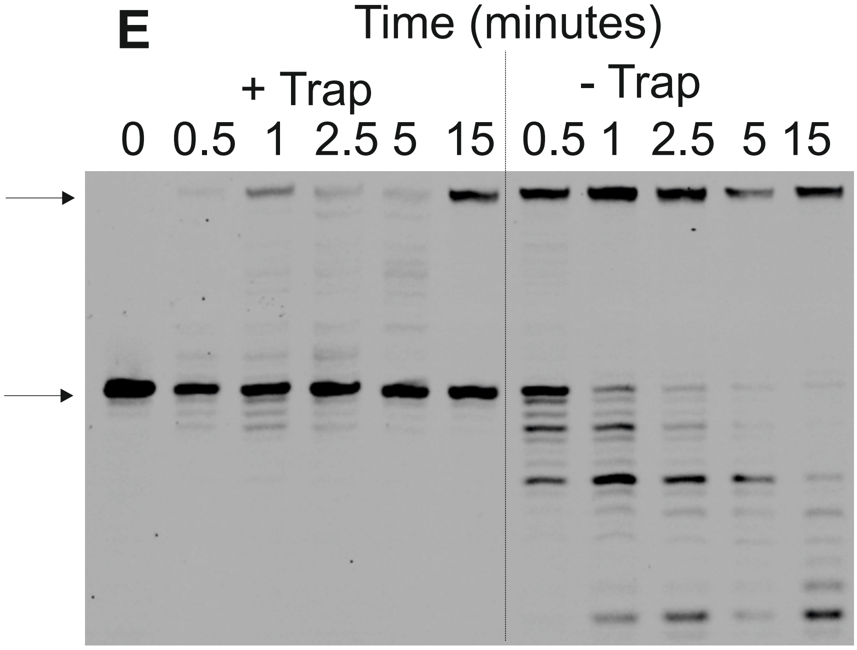

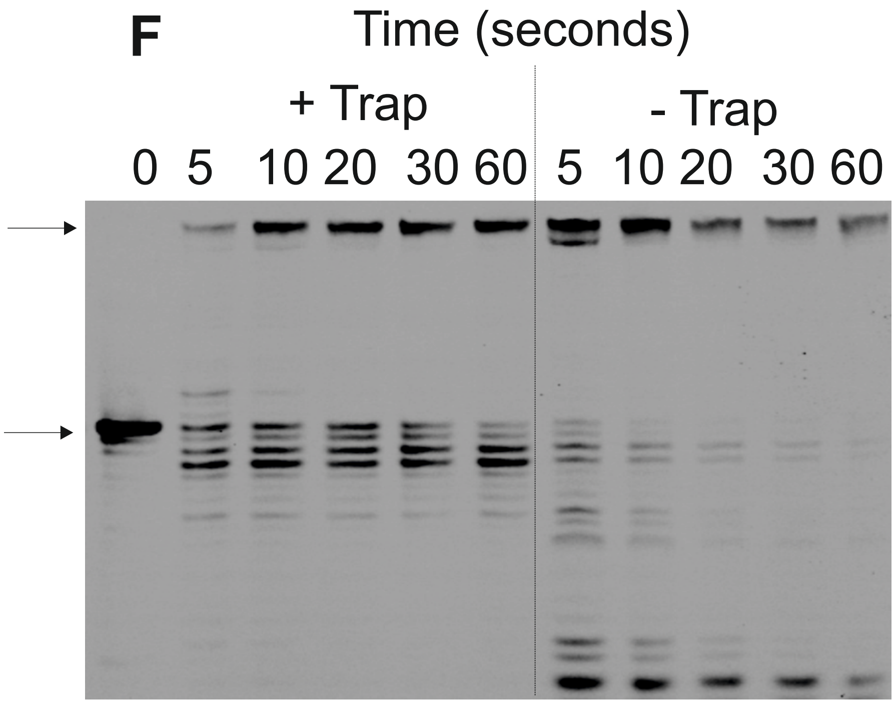

Supplement: Supplementary file 1 [file Presentation1.PDF]
